# Cooperative Cellular Uptake and Activity of Octaarginine Antisense Peptide Nucleic Acid (PNA) Conjugates

**DOI:** 10.3390/biom9100554

**Published:** 2019-10-01

**Authors:** Mahdi Ghavami, Takehiko Shiraishi, Peter E. Nielsen

**Affiliations:** Department of Cellular and Molecular Medicine, University of Copenhagen, Blegdamsvej 3, DK-2200 Copenhagen N, Denmark; mahdigh@sund.ku.dk (M.G.); shiraishiplant@gmail.com (T.S.)

**Keywords:** antisense, PNA delivery, octaarginine conjugate, cell-penetrating peptides, self-assembled nanoparticles

## Abstract

Cellular uptake and antisense activity of d-octaarginine conjugated peptide nucleic acids (PNAs) is shown to exhibit pronounced cooperativity in serum-containing medium, in particular by being enhanced by analogous mis-match PNA–cell-penetrating peptide (PNA–CPP) conjugates without inherent antisense activity. This cooperativity does not show cell or PNA sequence dependency, suggesting that it is a common effect in cationic CPP conjugated PNA delivery. Interestingly, our results also indicate that Deca-r8-PNA and r8-PNA could assist each other and even other non-CPP PNAs as an uptake enhancer agent. However, the peptide itself (without being attached to the PNA) failed to enhance uptake and antisense activity. These results are compatible with an endosomal uptake mechanism in which the endocytosis event is induced by multiple CPP–PNA binding to the cell surface requiring a certain CPP density, possibly in terms of nanoparticle number and/or size, to be triggered. In particular the finding that the number of endosomal events is dependent on the total CPP–PNA concentration supports such a model. It is not possible from the present results to conclude whether endosomal escape is also cooperatively induced by CPP–PNA.

## 1. Introduction

The DNA mimic peptide nucleic acid (PNA) has shown promising properties for development of efficient mRNA splice modulation antisense agents in a range of cellular systems and also in in vivo studies [1,2,3,4,5,6,7]. PNAs exhibit favorable characteristics compared to most oligonucleotides with regard to hybridization efficiency and biostability [8]. However, like antisense agents in general PNAs are not readily taken up by eukaryotic cells. Commonly, cationic lipid complexes are employed as effective vectors to deliver negatively charged antisense oligonucleotides (ASOs), while delivery of PNA using cationic lipid formulations requires conjugation to lipophilic or anionic domains, or prehybridization to a carrier oligonucleotide (e.g., DNA) [9,10]. Furthermore, use of cationic lipids in vivo is extremely limited because of general toxicity [11]. Alternatively, cellular uptake can be dramatically enhanced by chemical conjugation to cell-penetrating peptides (CPPs) (e.g., Tat, Penetratin, or oligoarginines) [12]. Although cellular internalization of CPPs has been intensely investigated, the mechanism(s) is (are) far from fully understood. Nonetheless, it is generally accepted that endocytosis, in particular macropinocytosis, is an important route of cellular entry of cationic CPPs [13,14,15,16,17]. The uptake pathway for CPP-mediated delivery depends on the size and physicochemical nature of the cargo molecule [18,19,20]. However, it is known that a major fraction of the CPP-conjugate accumulates in endosomal compartments without direct access to the cytosol or nucleus. Therefore, high concentrations (micromolar) of CPP-conjugated PNA are typically required in order to obtain significant biological responses [14,15], making inefficient endosomal escape a major limitation encountered for CPP-PNA cellular delivery. Thus several enhancers such as chloroquine, calcium ions, or lipophilic photosensitizer have been identified for inducing more efficient endosomal release [21,22,23]. However, it has also been reported that endosomal leakage can be enhanced by increasing the concentration of some cationic CPPs (especially arginine-rich) inside the endosomes [24]. In this study, cooperative effects of octaarginine (r8), and decanoyl-octaarginine (Deca-r8) [25] conjugated PNA on cellular uptake and antisense activity in serum-containing medium are demonstrated using the HeLa pLuc/705 cellular splice correction assay, fluorescence (confocal) microscopy, as well as flow cytometry, and evidence is presented indicating that the cellular uptake may be facilitated by nanoparticle formation in the presence of serum.

## 2. Materials and Methods

### 2.1. Peptide Nucleic Acid (PNA) Synthesis

PNAs were synthesized using the standard Boc method as described previously [26]. CPP peptides were linked to the N-terminal of PNAs using lysine or cysteine residues as the linker. This was performed via continuous solid phase synthesis. The deca fatty acid was conjugated to the ε-amino group of lysine. The cleavage was performed by trifluoroacetic acid (TFA)/ trifluoromethanesulfonic acid (TFMSA), and PNAs were purified by high-performance liquid chromatography (HPLC). The fluorescent dye (AF555) was coupled to an incorporated cysteine residue via the maleimide-thiol reaction. PNAs were purified again by HPLC, and characterization was conducted by matrix assisted laser desorption ionization—time of flight (MALDI-TOF) mass spectrometry.

### 2.2. Cell Culture

The cell lines, HeLa pLuc/705, PC-3, and P53R were obtained from Gene Tools, Sigma-Aldrich (Copenhagen, Denmark), and ATCC, respectively. The cells were grown in RPMI-1640 medium supplemented with 100 U/mL penicillin, 100 μg/mL streptomycin, 1% glutamax, and 10% fetal bovine serum (FBS), all form Thermofisher Scientific (Roskilde, Denmark), at 37 °C with 5% CO_2_. This supplemented cell medium will be referred to as a full medium. 

### 2.3. PNAs Antisense Activity and Toxicity

The HeLa pLuc/705 cells were seeded in 96-well (1.0 × 10^4^ cells/well) or in 24-well (1.0 × 10^5^ cells/well) plates 20 h before PNA treatment in 100 μL/well or 500 μL/well of full medium, respectively. Solutions of PNA and enhancer were prepared at 10× the final concentration in full medium and 10 μL (for 96 well) or 50 μL (for 24 well) of it was added to each well containing cells and mixed well. The cells were incubated for 19–20 h. and subjected to luciferase activity analysis and cytotoxicity assay using the Bright-Glu Luciferase assay system and Cell Titer proliferation ATP assay (both from Promega, Madison, WI, USA), respectively. These were performed according to the company’s protocols. Briefly, the cells were lysed with 60 μL Passive Lysis Buffer (Promega) of which 50 μL was used for the Luciferase assay and 10 μL was used for the ATP assay. Luminescence readings were normalized against ATP measurement of each sample and are presented as relative luminescent unit per ATP (RLU/ATP).

### 2.4. Reverse Transcriptase Polymerase Chain Reaction

For the reverse transcriptase polymerase chain reaction (RT-PCR) analysis of mRNA splicing the cells were lysed with 0.1 mL/well of Passive Lysis Buffer (Promega). Total RNA was then extracted using the RNeasy Mini Kit (Qiagen, Copenhagen, Denmark) and subjected to RT-PCR analysis. A total of 3 ng of RNA was utilized for each RT-PCR reaction (20 μL). RT-PCR was conducted by the OneStep RT-PCR kit (Qiagen) based on the manufacturer’s instruction. The forward and reverse primers used for RT-PCR are as follows: forward primer, 5′-TTG ATA TGT GGA TTT CGA GTC GTC-3′; reverse primer, 5′-TGT CAA TCA GAG TGC TTT TGG CG-3′. The products were analyzed on a 2% agarose gel in TBE buffer and visualized by ethidium bromide staining. Then the DNA band intensities in gel images were quantified by UN-SCAN-IT software version 5.1 (Silk Scientific, Orem, UT, USA). 

### 2.5. Fluorescence and Confocal Microscopy

For fluorescence microscopy studies, HeLa pLuc/705cells were seeded in 96-well plates at a density of 1.0 × 10^4^ cells/well the day before PNA treatment. The fluorophore-PNA solution was prepared at 1.25 μM (10x the final concentration) in full medium, the non-labeled PNA acting as enhancer was added to the solution at 10× final concentrations. Ten μL of PNA solution was added to the cells in each well containing 100 μL full medium and incubated for 2 h. Hoechst 33342 was added to each well (at a final concentration of 3 μg/mL) in order to stain the nucleus 10 min before microscopy analysis. The medium was removed by aspiration, and the cells were washed three times with the pre-warmed (37 °C) full medium. The images were obtained using a green filter for AF555 and a blue filter for Hoechst 33342, and the two images merged using Fiji ImageJ open source software [27].

For fluorescence confocal microscopy, HeLa pLuc/705cells were plated on μ-Slide 8 well Glass Bottom (ibidi, Gräfelfing, Germany) at a density of 3.0 × 10^4^ cells/well in full medium the day before PNA treatment. The PNA solutions were prepared in 300 μL of full medium, and the cell medium was replaced with this solution. The concentration of AF-PNA was 0.125 μM. Non-labeled PNA was added to the solution at different concentrations as an enhancer. The treatment time was 2 h, and Hoechst 33342 was added to each well (3 μg/mL final concentration) 10 min before being subjected to the microscopy study. The medium was removed, and the cells were gently washed three times with pre-warmed full medium. The cells were analyzed using an LSM 750 confocal scanning microscope (Zeiss, Oberkochen, Germany), and the images were analyzed with ZEN 2.3 SP1 software (Zeiss) and Fiji ImageJ software. Uptake events were quantified by adjusting the fixed threshold (10–100) for all images and then using “Analyze particles” function of Fiji ImageJ to count the number of uptake events.

### 2.6. Flow Cytometry

HeLa pLuc/705 cells were seeded in 24 well plates at 1.0 × 10^5^ cells/well the day before PNA treatment. PNA solutions were prepared at 10× concentration as described in the PNAs antisense activity and toxicity section. 50 μL of PNA solution was added to wells containing 500 μL full medium and mixed well. Cells were incubated for 4 h, and then washed three times with pre-warmed full medium. Cells were detached from plates by gently pipetting with phosphate buffered saline (PBS) containing 1% bovine serum albumin (BSA). Cells were then filtered with a cell strainer (70 μm, BD Falcon) and subjected to flow cytometric analysis (30,000 events for each sample) using Accuri C6 Personal Flow Cytometry (BD Biosciences, Lyngby, Denmark). The mean fluorescence signal was calculated by FCS Express 6 Flow Cytometry Software (De Novo Software, Glendale, CA, USA).

### 2.7. Cellular Uptake by Fluorescence Reading in 96-Well Plates

Exponentially growing cells (HeLa pLuc/705, PC-3 or P53R) were plated in 24-well plates at a density of 1.0 × 10^5^ cells/well the day before PNA treatment. After 19-20 h incubation, the cells were treated with PNA solutions prepared as described in the Flow Cytometry section. The cells were incubated for 4 h with PNA, and Hoechst 33342 (3 μg/mL final concentration) was added to the cells 10 min before transfection was complete. Cells were washed trice with pre-warmed full medium, and collected by pipetting cells in 200 μL PBS containing 1% BSA. 100 μL of each sample was placed in 96-well plates, and the fluorescent signals were measured using the Synergy H1 plate reader (BioTek, Winooski, VT, USA). The signal from the PNAs in each well was normalized against the signal from Hoechst DNA staining and is presented as relative fluorescence per cell (FL/Cell).

### 2.8. Size of Self-Aggregated Particles

Dynamic light-scattering (DLS) measurements were performed by a Zetasizer Nano ZS (Malvern Instruments, Westborough, MA, USA) and they were carried out with a 633 nm laser and 173° detection optics in disposable sizing cuvettes. The r8-PNA at the concentration of 1 μM was used in the absence and presence of 1, 2, and 4 μM enhancer (r8-PNA_mm_) in full medium according to the antisense activity experiment. The same procedure was also performed in serum-free condition using RPMI-1640 without FBS. 

### 2.9. Characterization of PNA–Protein Interaction

Full medium and RPMI-1640 (without FBS) were centrifuged at 20,000× *g* for 40 min at 4 °C to remove any pre-existing protein aggregates. Then 5 μl of r8-PNA (200 μM) or 5 μl of r8-PNA (200 μM) plus 20 μl of enhancer (r8-PNA_mm_) (200 μM) were added to 1000 μl of pre-centrifuged cell medium (with and without FBS). Samples were incubated at 37 °C for one hour, and then centrifuged at 20,000× *g* for 40 min at 4 °C. The supernatant was removed from each tube and 1000 μl PBS was added to each as a washing step followed by centrifugation at 20,000× *g* for 40 min at 4 °C. After three washing steps, 13 μl water, 5 μl protein-loading buffer (NuPAGE LDS Sample Buffer, Thermofisher Scientific, Roskilde, Denmark), and 2 μl reducing agent (NuPAGE Sample Reducing Agent, Thermo Fisher Scientific) were added to each tube and after mixing and incubating at 70 °C for 10 min, the samples were run on 4–12% precast midi gel (NuPAGE Bis-Tris Gels, Thermo Fisher Scientific) at 200 volt for 30 min. Subsequent silver gel staining was used to detect proteins on the gel.

## 3. Results

### 3.1. Cooperative PNA Activity 

The intracellular activity of PNAs was measured using the well-established HeLa pLuc/705 cell system which is based on antisense induced pre-mRNA splicing correction of a luciferase mRNA, and consequently luciferase induction [28]. The HeLa pLuc705 cell line harbors a luciferase gene that is interrupted by a thalassemic β-globin intron 2 which contains a cryptic splice site resulting in the retention of part of the intron in the luciferase pre-mRNA thereby producing an inactive luciferase protein. The luciferase pre-mRNA splicing can be corrected by blocking the cryptic splice site using PNA as an antisense agent thus resulting in synthesis of functional luciferase [12,28]. The antisense PNA oligomer of the PNA-arginine conjugates has previously been validated by a variety of delivery methods, as well as corresponding two mismatch negative controls (Table 1) for validating the antisense mode of action [3,21,25]. A previous study in serum-free medium during delivery has shown that enhanced cellular antisense activity can be obtained by attaching a lipid domain to CPP–PNAs forming Deca-r8-PNAs (CatLip PNAs) [25].

**Figure 1:**
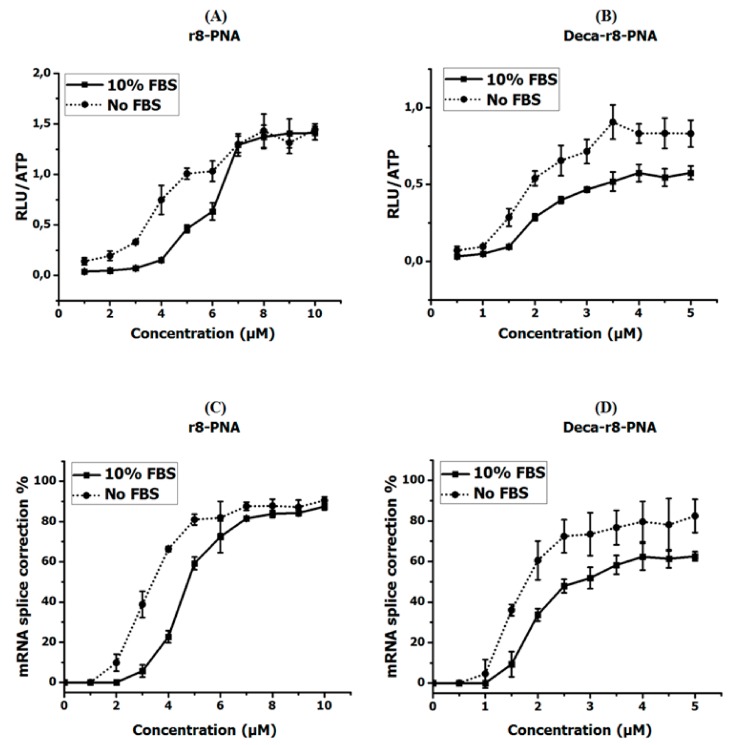
Antisense activity of r8- and Deca-r8- conjugated PNAs in the absence and presence of serum proteins. (**A**,**B**) show the luciferase activity after PNA treatment and (**C**,**D**) depict the corresponding mRNA splice correction after PNA treatment. The PNA treatment time was 4 h then PNA containing medium was changed with full medium and cells were incubated overnight. The luciferase activity is normalized by live cells (RLU/ATP) (see Figure S1). Each data set represents the mean ± standard deviation (SD) of triplicate experiments.

The present results (Figure 1) show that in serum containing as compared to serum free medium the antisense activity of both r8-PNA and Deca-r8-PNA is only slightly reduced, and that in all cases a non-linear dose response of luciferase activity and mRNA splice correction is seen at lower PNA concentrations which could indicate molecular cooperativity of cellular uptake and/or splice correction.

**Figure 2:**
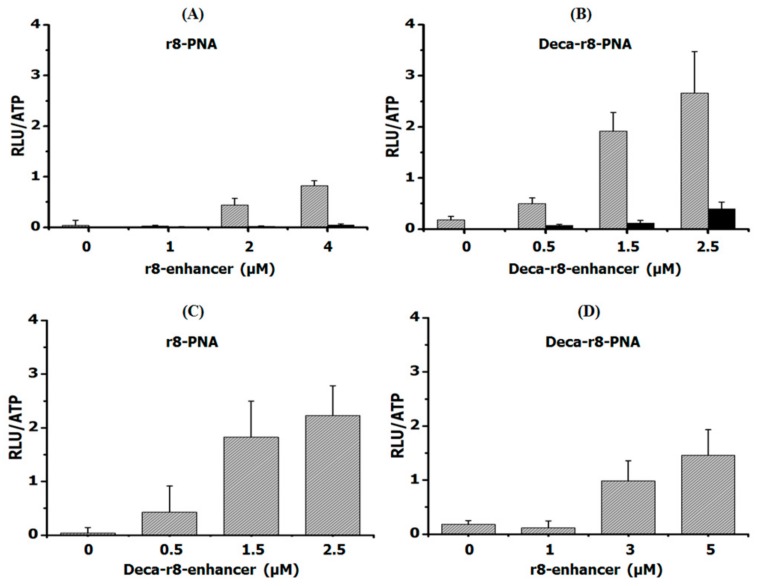
Antisense enhancer effect of different PNA conjugates. (**A**) r8-PNA at a concentration of 1 μM was used for all samples and r8-PNA_mm_ at different concentrations was added as enhancer. (**B**) Deca-r8-PNA at 0.5 μM was used and Deca-r8-PNA_mm_ at different concentrations was added as enhancer. (**C**) r8-PNA at 1 μM was used for all conditions and Deca-r8-PNA_mm_ at different concentrations was added as enhancer. (**D**) Deca-r8-PNA at 0.5 μM was used and r8-PNA_mm_ at different concentrations was added as enhancer. The treatment time was 20 h and luciferase activity is normalized by live cells (RLU/ATP). Each data set represents the mean ± SD of the triplicate experiment. Grey bars show active PNA plus enhancer, while black bars in (**A**,**B**) show enhancer alone.

If a cooperative mechanism independent of target sequence recognition is involved, the uptake and biological activity of a CPP-PNA should be increased by addition of an analogous that does not bind the mRNA target. Thus we investigated the effect of a mismatch CPP-PNA, which by itself does not affect luciferase activity, as a possible “enhancer” for cellular uptake (endocytosis/endosomal escape). The results (Figure 2A,B) reveal that the biological activity of the PNA in terms of luciferase activation was indeed increased in a dose dependent manner by the enhancer, and the antisense mechanism responsible for the activation was confirmed by RT-PCR showing a corresponding increase in the level of corrected mRNA in the cells (Figure S2). Indeed, a replot of the results of Figure 2B using the total CPP-PNA concentration shows that the activity increases with total CPP-PNA concentration with a slope close to 1 (Figure S2E). This is compatible with an apparent bi-molecular reaction resulting in an exponential dependence of the antisense activity (aa) on concentration of PNA if the match had been added instead of the mismatch (aa = k × [CPP-PNA_match_]^2^), thereby supporting (bimolecular) cooperativity as hinted at by the data in Figure 1. It is also noteworthy that lower concentrations of the lipophilic Deca-r8-PNA_mm_ compared to r8-PNA_mm_ yielded similar enhancement. We also performed “cross-carrier” experiments in which the mismatch Deca-r8-PNA_mm_ was added to r8-PNA, and vice versa. These results likewise show that upon addition of another antisense inactive peptide-PNA conjugate, the PNA antisense activity was still enhanced (Figure 2C,D and Figure S2). Thus by adding mismatch Deca-r8-PNA (final concentration of 1.5 μM) as an enhancer to r8-PNA (final concentration of 1 μM) the antisense activity was increased up to 10-fold based on luciferase activity. While the addition of mismatch r8-PNA (final concentration of 3 μM) as an enhancer to the Deca-r8-PNA (final concentration of 0.5 μM) the luciferase activity was only increased up to 5-fold (Figure 2). This indicates that Deca-r8-PNA is the more effective enhancer of the cellular uptake and/or endosomal release process.

### 3.2. Cellular Uptake of PNA Using Fluorescence Microscopy 

**Figure 3:**
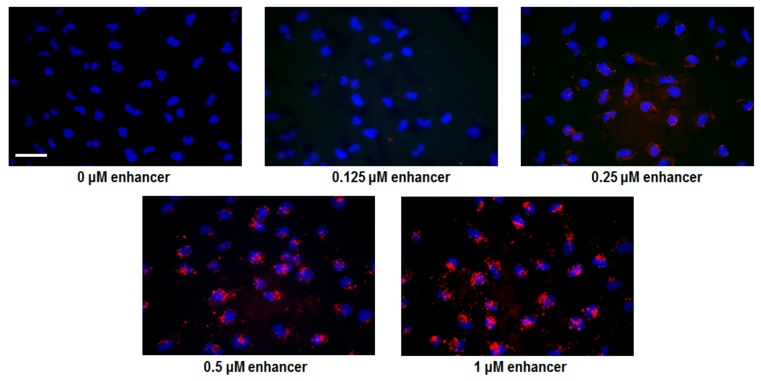
Uptake enhancer studies by fluorescence microscopy. Deca-r8-AF-PNA 5103 (red) was used as the labeled PNA at a concentration of 0.125 μM in all experiments. Deca-r8-PNA 2784 (non-labeled PNA) was added at different concentrations as uptake enhancer. The cells were incubated for 2 h at 37 °C after adding PNA to the cells. Hoechst 33342 was added as a nuclear stain (blue) 10 min before capturing the images. The scale bar is 30 μm.

Microscopy studies using fluorophore labeled PNA were conducted in order to directly address cellular uptake enhancement/cooperativity. Live cell imaging was performed because the endosomal compartments of cells are damaged upon fixation, resulting in the artificial release of CPP-PNA from endosomes (Figure S3) [24]. Initially an AlexaFluor555 (AF555) derivative of the most active Deca-r8-PNA (Deca-r8-AF-PNA) was chosen for monitoring uptake, and non-labeled Deca-r8-PNA was used as the enhancer. A clear increase in intracellular fluorescence signal upon addition of enhancer was observed (Figure 3), indicating that the Deca-r8-PNA could efficiently increase cellular uptake of Deca-r8-AF-PNA. The results also clearly illustrate that the internalization of PNA increased with enhancer concentration, and this was not dependent on PNA sequence as using PNA of a different nucleobase sequence resulted in a similar effect (Figure S4).

**Figure 4:**
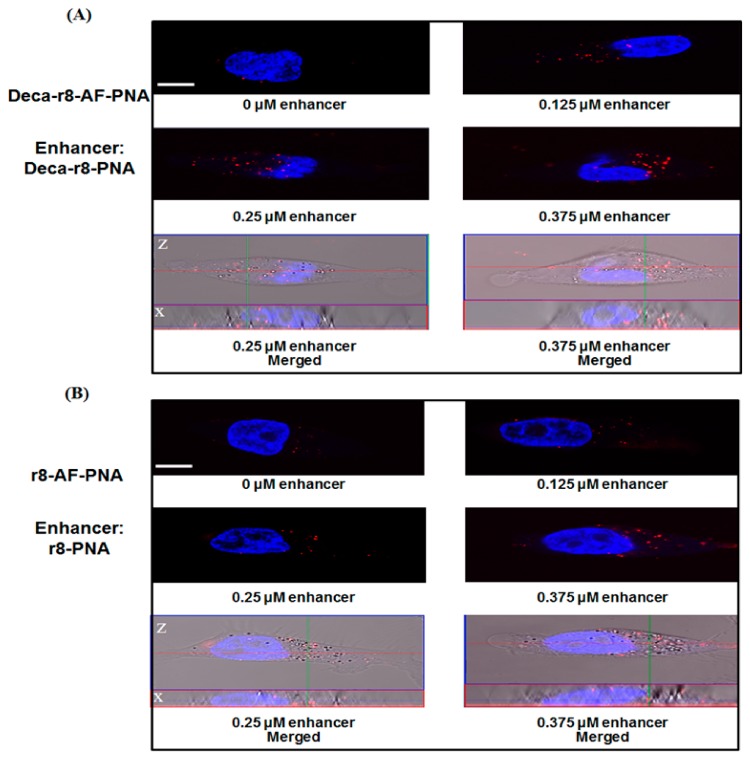
Confocal microscopy images of cells after PNA treatment for 2 h. The concentration of (**A**) Deca-r8-AF-PNA and (**B**) r8-AF-PNA was 0.125 μM and the enhancer Deca-r8-PNA (**A**) or r8-PNA (**B**) was added to the cells at different concentrations. The nuclei were stained with Hoechst 33342 (blue) and signals from PNA-AF are red. Hoechst 33342 was added 10 min before taking images. The scale bar is 5 μm.

More detailed information of the mechanism of PNA internalization and enhancement was achieved by confocal microscopy using both Deca-r8-AF-PNA (Figure 4A) and r8-AF-PNA (Figure 4B). It can be seen that PNA is entrapped in vesicles (endosomes) inside the cell (Figure 4 and Figure S6), and that the number of vesicles containing PNA increases in the presence of the enhancer in a dose dependent manner. Thus the addition of enhancer results in an increase in the number of PNA endocytotic events (rather than the magnitude of each event), thereby corroborating a cooperative mechanism of cellular endocytotic uptake per se. The results do not address whether the total number of endocytotic events increases as only AF-PNA containing endosomes are detected. In addition, the more lipo- and amphiphilic Deca-r8-AF-PNA exhibits stronger antisense activity (Figure 1) as well as uptake enhancer potency than the simple oligoarginine conjugate r8-AF-PNA. However, the AlexaFluor derivatives themselves are slightly less potent antisense agents compared to their unmodified homologoues (Figure S5).

### 3.3. Cellular Uptake Quantification by Flow Cytometry 

**Figure 5:**
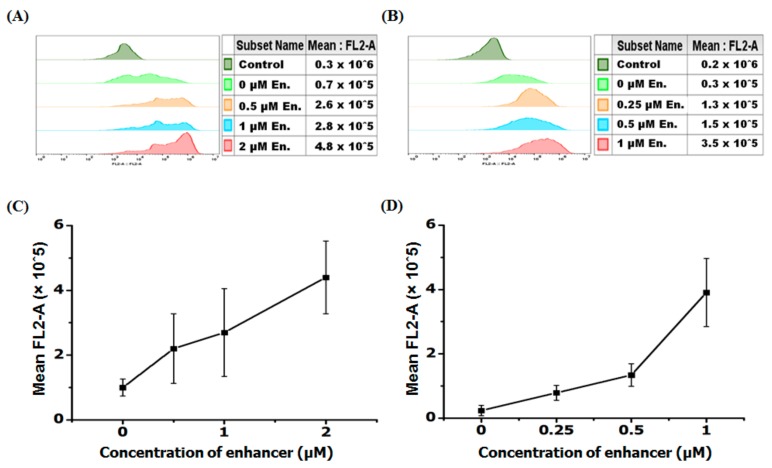
Enhancer effect of PNA uptake using flow cytometry. Mean cellular fluorescence at varying enhancer concentration. (**A**) r8-AF-PNA at the concentration of 0.5 μM was used for all cells and r8-PNA at different concentrations was added as enhancer. (**B**) Deca-r8-AF-PNA at the concentration of 0.25 μM was used and Deca-r8-PNA at different concentrations was added as enhancer. The transfection time was 4 h and the cells were washed 3 times before analysis by flow cytometry. (**C**,**D**) Mean FL2-A figures from flow cytometry histograms (**A**,**B**) are used to draw graphs, respectively. Control cells without PNA are used as background and each data set represents the mean ± SD of triplicate experiment.

In order to obtain a more accurate quantification of the PNA uptake as well as extending the study to other cell types, flow cytometry experiments were carried out using the fluorophore labeled r8-AF- and Deca-r8-AF-PNAs (Figure 5) as a semi-quantitative analysis of cellular uptake. The results confirm that the addition of enhancer results in a very significant increase in the amount of PNA taken up by the HeLa pLuc/705 cells, and again the Deca-r8-AF-PNA is the more potent compared to r8-AF-PNA. The uptake of Deca-r8-AF-PNA at a concentration of 0.25 μM with 1 μM enhancer added is similar to the uptake of r8-AF-PNA at a concentration of 0.5 μM with 2 μM enhancer (Figure 5C,D). Analogous enhancement was found for two other cell lines, PC-3 (prostate cancer cells) and p53R (colon cancer cells) (Figure S7) and, therefore, the phenomenon is not limited to a specific cell line.

### 3.4. Enhancing Effect of Deca-r8-PNA on Non-Peptide PNA Derivatives

**Figure 6:**
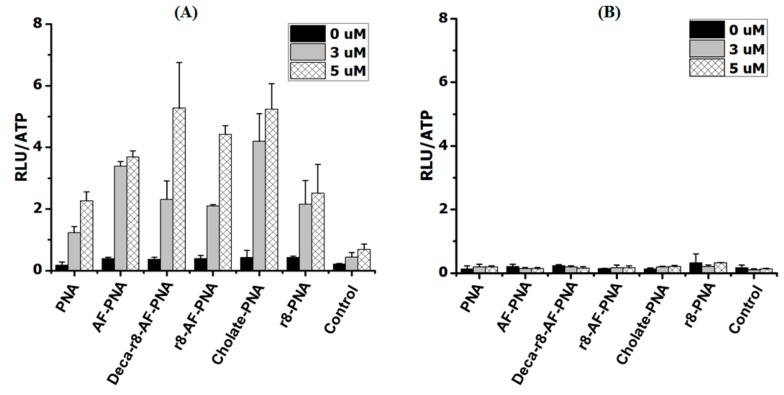
The ability of Deca-r8-PNA or Deca-r8 peptide to assist cellular activity of other PNAs. The antisense PNAs were used at the concentration of 1 μM and (**A**) Deca-r8-PNA_mm_ or (**B**) Deca-r8 peptide was added at 3 or 5 μM as enhancer. Cells were incubated for 20 h. Controls show the activity in the presence of Deca-r8-PNA_mm_ or Deca-r8 peptide alone at different concentrations. The activity is normalized by live cells (RLU/ATP) please refer to Figure S8. Each data set represents the mean ± standard deviation (SD) of triplicate experiments.

The results show that both Deca-r8-PNA and r8-PNA exhibit cooperative cellular uptake. In order to address any role of the PNA part in this phenomenon we asked whether the mismatch Deca-r8-PNA or the Deca-r8 peptide alone could enhance the cellular antisense activity of non-peptide PNA derivatives with limited inherent cellular activity. Surprisingly, we observed that the mismatch Deca-r8-PNA could significantly enhance the antisense activity of unmodified PNA as well as AF555 or cholate conjugated PNA (to a similar degree), whereas no enhancement was seen using the Deca-r8 peptide alone (Figure 6). These results clearly show that enhancement does not require that the antisense PNA itself is conjugated to a CPP or another uptake ligand although lipophilic ligands (such as AF555 or cholate) appear to increase activity (Figure 6; 3 μM enhancer). Furthermore, the PNA part of the enhancer is instrumental for the enhancement activity, Thus the combined results would indicate that the enhancer effect is connected to non-covalent, partly hydrophobic interactions between the active PNA(-conjugate) and the enhancer (PNA). This feature may be related to uptake promoted by the “Pepfect”-type long chain fatty acid-CPP conjugates, which are very efficient for oligonucleotide delivery [29], or to the uptake and activity of self-assembled nano-aggregates of lipophilic bicycloguanidinium-PNA conjugates [30].

### 3.5. Nano-Aggregate/Particle Formation in the Presence of Serum 

**Figure 7:**
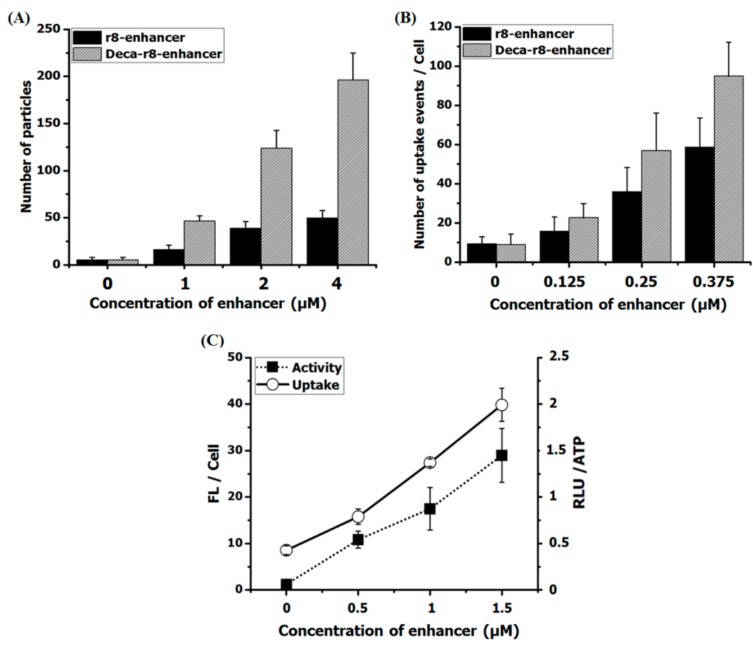
Linear relation between number of formed particles, uptake events and activity of PNA. (**A**) The AF-PNA at the concentration of 1 μM was prepared in full medium in the presence of different concentrations of enhancers and the number of particles was evaluated form fluorescent images. (**B**) The AF-PNA cellular uptake at the concentration of 0.125 μM in the presence of enhancers at different concentrations (please also refer to Figure 4). The number of particles (**A**) and uptake events (**B**) are calculated by “Fiji Image J” via the function of “analyze particles” with a threshold of 10–100 for all samples from fluorescent images. (**C**) PNA antisense activity and cellular uptake were evaluated for AF-PNA at the concentration of 0.5 μM with different concentration of enhancer (Deca-r8-PNA_mm_). Each data set represents the mean ± SD of triplicate experiment.

**Figure 8:**
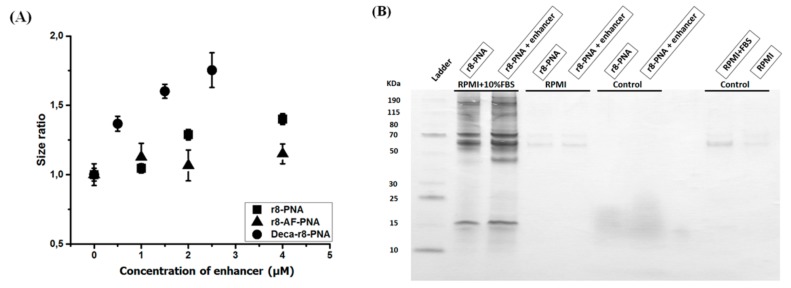
Particle size and formation in the presence of 10% FBS. (**A**) Particle size increases in a dose dependent manner. By adding different enhancers the size of formed particles increases and is reliant to various PNA-conjugates. The sizes are measured by dynamic light scattering and each data set represents the mean ± SD of triplicate experiment. The size without enhancer addition is 150 nm. (**B**) The r8-PNA (1 μM) with and without r8-enhancer (4 μM) were interacted with cell medium (RPMI-1640) at 37 °C for one hour in the presence and absence of serum proteins (10% FBS). After sample centrifugation followed by three subsequent washing steps with phosphate-buffered saline (PBS) and additional centrifugations, samples were run on SDS-polyacrylamide gel to detect any possible protein bands incorporated with PNA nano-aggregations. r8-PNA with and without r8-enhancer were run as controls (without any centrifugation), and RPMI with and without serum proteins were used as additional controls (with all centrifugations and washing steps).

Recent studies have also implicated nanoparticle formation as an integral mechanism in the cellular antisense activity of phosphordiamidate morpholino oligomers (PMOs) [31]. In connection with the fluorescence uptake studies described above indications of aggregation of the fluorophore-labeled PNA in the cell culture was observed. This was most significant at higher concentrations (2–10 μM) and depending on the specific PNA conjugate (Figures S9 and S10); and the effect was more pronounced for the decanoyl-arginine conjugate. In addition, it was observed that the aggregation of the fluorophore PNAs was increased by addition of the non-labeled enhancer PNA r8-PNA and deca-r8-PNA conjugates in terms of number of particles formed (Figure 7A and Figure S11), and again more pronounced for the lipophilic decanoyl derivative. Most interestingly, fluorescence microscopy analysis of the cellular uptake under these conditions revealed a direct correlation between the number of particles and the number of cellular uptake (endocytosis) events (Figure 7A,B). Finally, the increased aggregation and cellular uptake was also directly correlated with increased cellular antisense activity (Figure 7C). These results strongly suggest that the observed cooperative cellular antisense activity may at least in part be ascribed to increased cellular uptake in terms of endocytosis events driven by PNA-conjugate aggregation/nanoparticle formation. In order to further substantiate and characterize these particles also in the case of PNAs not containing a fluorophore (which may enhance aggregation/particle formation), we resorted to dynamic light scattering analysis using the r8-PNA, r8-AF-PNA and deca-r8-PNA. From these results (Figure S12) we conclude that well-defined 100-200 nm nanoparticles were detected for all three PNA conjugates in cell culture medium, but only in the presence of serum. Furthermore, the particle size increased in a dose dependent way upon addition of enhancer, and this increase was most pronounced for deca-r8-PNA and to a lesser extent for r8-AF-PNA (Figure 8A). Due to the apparent requirement for serum in the formation of uniform (nano)particles, we speculated that serum proteins could play a role either for nanoparticle formation or their stability. To address possible involvement of proteins, these nanoparticles were isolated by centrifugation in the presence and absence of serum and analyzed by SDS-polyacrylamide gel electrophoresis for protein content. The results shown in Figure 8B, indeed confirm that serum proteins were pelleted with the nanoparticles thereby strongly implicating protein association or integration.

## 4. Discussion

It is generally accepted that endosomal uptake and subsequent release pathways are instrumental for cellular uptake mediated by cell-penetrating peptides (CPPs) including oligo-arginines, and that the endosomal uptake may occur via several mechanisms, including clathrin-coated pits, caveolin-dependent endocytosis, clathrin/caveolin independent endocytosis, and macropinocytosis [32]. Likewise, most nanoparticle and lipofection delivery systems exploit endocytosis for cellular delivery [33,34] as do self-assembling CPPs [35]. Recently it was also reported that cellular uptake of some PMO antisense agents may occur by self-assembly in nanoparticles that are taken up by caveolin-dependent endocytosis via the scavenger receptor class A1 [31]. In addition, it has been shown that the cell surface affinity of cationic CPPs is reduced by attachment to macromolecules, but may be alleviated through cargo self-assembly [36]. Furthermore it has been found that self-assembly into nanoparticles is important for oligonucleotide uptake and gene transfer mediated by oligoarginines [31,37], and likewise the cellular uptake oligo(bicycloguanidinium)-PNA conjugates [30] is correlated to their propensities for self-assembly and/or aggregation. Thus ample studies have demonstrated that (nano)particle formation can enhance or even be a prerequisite for efficient endosomal uptake.

The present data clearly support the assertion that cellular uptake and antisense efficacy of PNA–CPP conjugates—in casu octaarginine conjugates—in serum containing cell medium is also positively influenced by formation of nano-aggregates/particles induced by antisense inactive PNA-CPP conjugate enhancers. The results also show that the PNA part is required for enhancer activity, which is also further inceased by inclusion of lipophilic (decanoyl and alexafluor) moieties.

It is not clear which endocytosis pathway(s) is (are) involved in the cellular uptake of the particles but it could be related to analogous findings regarding receptor-mediated endocytosis, which have been reported to exhibit cumulative effects [38,39,40,41]. Also the recent finding of the possible involvement of the class A scavenger receptors in caveolin-dependent endocytosis cellular uptake of PMO nanoparticles may be relevant for this discussion [31]. However, more detailed cellular studies are required to resolve this (or these) mechanism(s). Nonetheless, these observations add another level of complexity to the properties and cell interactions of CPP conjugates.

## 5. Conclusions

The present results clearly demonstrate pronounced cooperativity in cellular uptake and antisense activity of d-octaarginine conjugated PNAs. This cooperativity does not show cell or PNA sequence dependency, suggesting that it is a common effect in cationic CPP-conjugated PNA delivery. Interestingly, our results also indicate that Deca-r8-PNA or r8-PNA could assist each other and even other PNAs as a transfection agent. However, the decanoyl-r8 peptide itself (without being attached to the PNA) failed to enhance antisense activity. Furthermore, uptake and antisense efficiency correlates with a number of endosomal uptake events, and in the presence of serum with nanoparticle formation. These results are compatible with an endosomal uptake mechanism in which the endocytosis event is induced by the CPP–PNA binding to the cell surface requiring a certain CPP density to be triggered, and which at least in the presence of serum (as relevant for the in vivo situation) also involves nanoparticle formation. It is not possible from the present results to conclude whether endosomal escape is also cooperatively induced by CPP. The discovered enhancement and cooperativity effects are mainly of mechanistic interest, but the observation of nanoparticle formation and possible involvement in cellular delivery may be exploited for the development of improved formulations for in vivo applications and development.

## Figures and Tables

**Table 1:** List of peptide nucleic acids (PNAs) and peptides.

PNA	Name	Sequence
2389	PNA	H-CCT CTT ACC TCA GTT ACA-NH_2_
5006	r8-PNA	H-(D-Arg)_8_-CCT CTT ACC TCA GTT ACA-NH_2_
2784	Deca-r8-PNA	H-(D-Arg)_8_-Lys(Decanoyl)- CCT CTT ACC TCA GTT ACA-NH_2_
3388	Deca-r8-PNA_mm_	H-(D-Arg)_8_-Lys(Decanoyl)- CCT CTG ACC TCA TTT ACA-NH_2_
5102	AF-PNA	Ac-Cys(AF555)-CCT CTT ACC TCA GTT ACA-NH_2_
5103	Deca-r8-AF-PNA	H-(D-Arg)_8_-Lys(Decanoyl)-Cys(AF555)-CCT CTTT ACC TCA GTT ACA-NH_2_
5104	r8-AF-PNA	H-(D-Arg)_8_-Cys(AF555)-CCT CTTT ACC TCA GTT ACA-NH_2_
5224	r8-PNA_mm_	H-(D-Arg)_8_-CCT CTG ACC TCA TTT ACA-NH_2_
2964	Cholate-PNA	Cholate-CCT CTT ACC TCA GTT ACA-NH_2_
5009	Deca-r8-AF-PNA2	Decanoyl-(D-Arg)_8_-Cys(AF555)-TCC AGA TGC CTT GGG-NH_2_
5012	Deca-r8-PNA2	Decanoyl-(D-Arg)_8_-Cys-TCC AGA TGC CTT GGG-NH_2_
Pep44	Deca-r8	Decanoyl-(D-Arg)_8_-Gly-NH_2_

## Supplementary Materials

The following are available online at https://www.mdpi.com/2218-273X/9/10/554/s1: Figure S1: Gels for mRNA splice correction and cell viability data, Figure S2: Splicing correction at mRNA level for the cooperative effect, Figure S3: The effect of cell fixation on uptake images, Figure S4: The antisense activity of conjugated PNAs with and without AlexaFluor, Figure S5: Uptake cooperativity of PNA2 studied by fluorescence microscopy, Figure S6: Confocal fluorescence image from cells, Figure S7: Uptake cooperativity in different cell lines, Figure S8: Cytotoxicity of PNAs in the presence of Deca-r8-PNA or Deca-r8 peptide, Figure S9: Aggregation of AlexaFluor labeled PNAs in full medium, Figure S10: Number of aggregated AlexaFluor labeled PNAs in full medium, Figure S11: Aggregation of AF-PNAs in full medium in the presence of r8-PNA or Deca-r8-PNA as enhancers, Figure S12: Size characterization of self-assembled nano-aggregations.
