# Supplementary material for: Cooperative Cellular Uptake and Activity of Octaarginine Antisense Peptide Nucleic Acid (PNA) Conjugates
Source: Biomolecules. 2019 Oct 1;9(10):554. doi: 10.3390/biom9100554 (PMC6843268; doi:10.3390/biom9100554)
Supplement: Supplementary file 1 [file biomolecules-09-00554-s001.pdf]

Supplementary Materials

Cooperative Cellular Uptake and Activity of Octaarginine Antisense Peptide Nucleic acid (PNA) Conjugates

Mahdi Ghavami, Takehiko Shiraishi and Peter E. Nielsen*

Department of Cellular and Molecular Medicine, Panum Institute, University of Copenhagen, Blegdamsvej 3, DK-2200 Copenhagen N, Denmark

*Correspondence to Peter E. Nielsen, Department of Cellular and Molecular Medicine, Panum Institute, University of Copenhagen, Blegdamsvej 3, DK-2200 Copenhagen N, Denmark, Tel: +45 35327762, Email: ptrn@sund.ku.dk

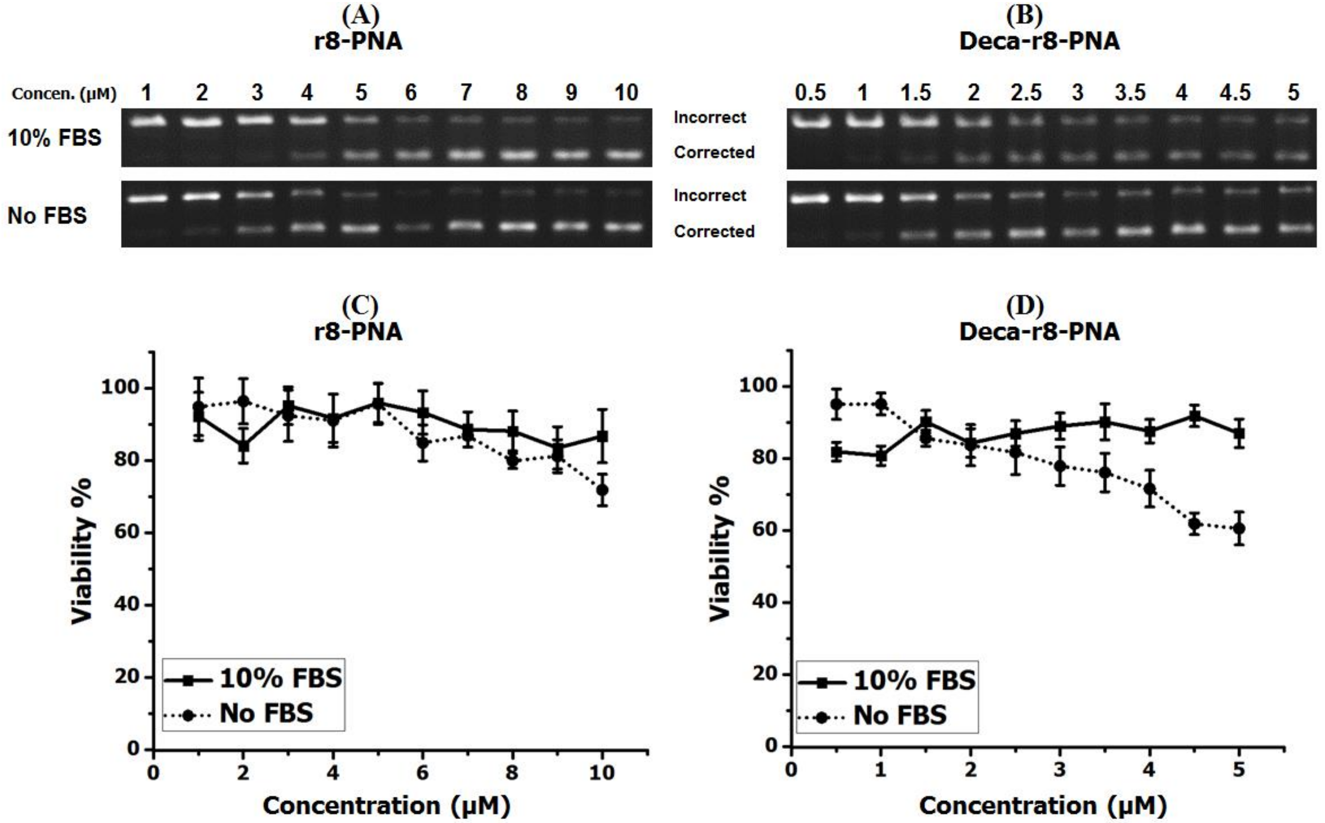

Figure S1. Gels for mRNA splice correction and cell viability. **(A)** & **(B)** depicts mRNA splice correction after PNA treatment in the absence and presence of serum. **(C)** & **(D)** shows cell viability. The PNA treatment time was 4 h then PNA containing medium was changed with full medium and cells were incubated overnight.

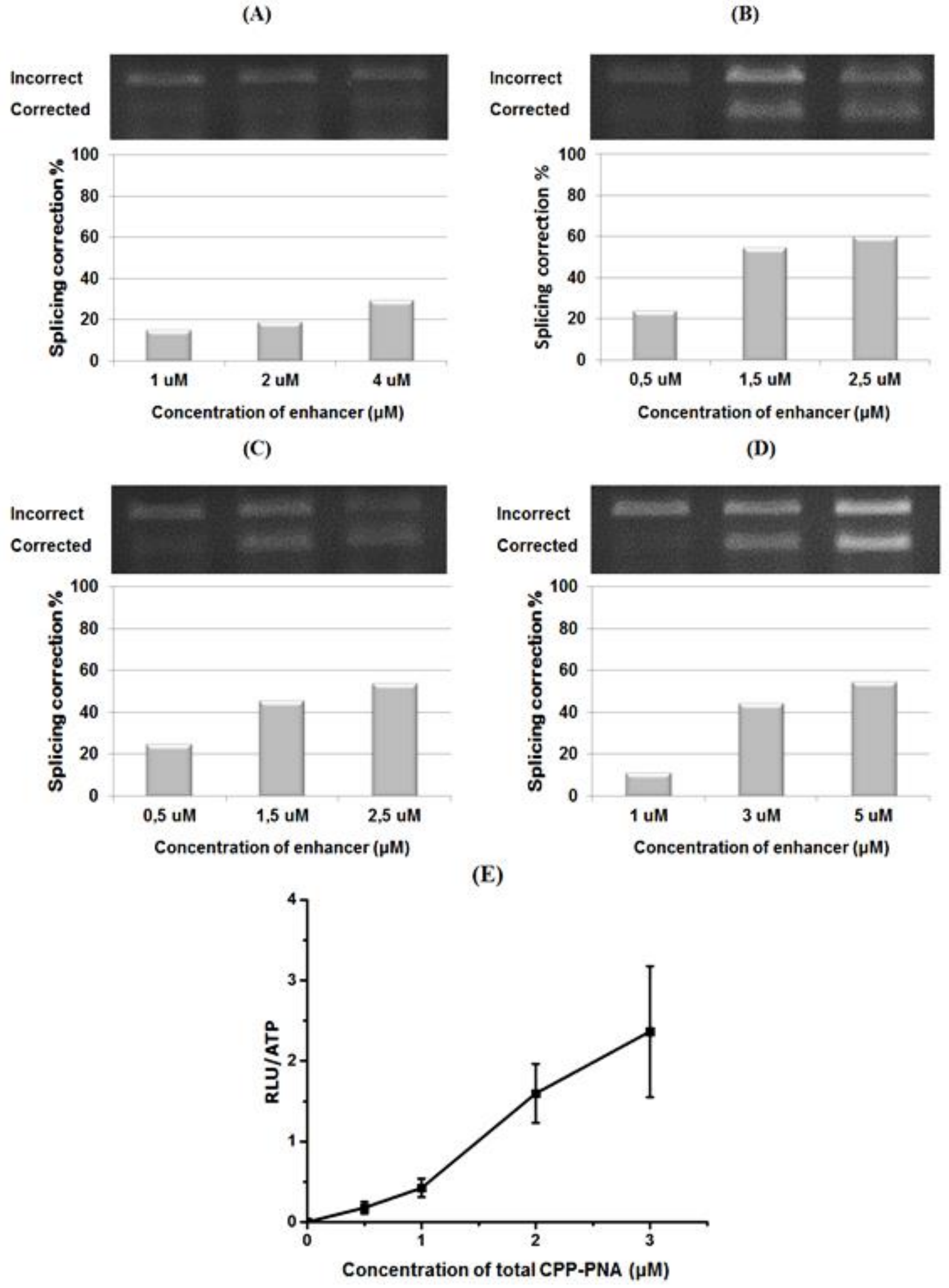

Figure S2. Splicing correction at mRNA level for the cooperativity effect. (A) r8-PNA at 1 µM was used for all conditions and r8-PNA$_{mm}$ at different concentrations was added as enhancer. (B) Deca-r8-PNA at 0.5 µM was used and Deca-r8-PNA$_{mm}$ at different concentrations was added as enhancer. (C) r8-PNA at 1 µM was used for all conditions and Deca-r8-PNA$_{mm}$ at different concentrations was added as enhancer. (D) Deca-r8-PNA at 0.5 µM was used and r8-PNA$_{mm}$ at different concentrations was added as enhancer. (E) The plot of total CPP-PNA (match + mismatch) concentration versus antisense activity.

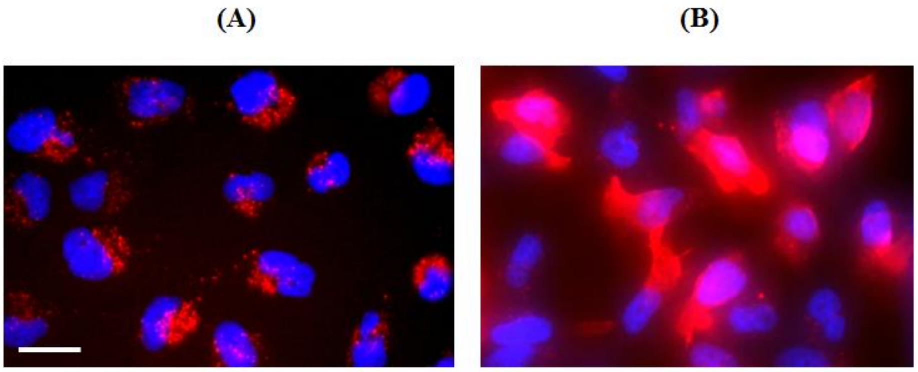

Figure S3. The effect of cell fixation on uptake images. HeLa pLuc/705 ells were incubated 2 h with Deca-r8-AF-PNA at 0.5 μM. (A) Live cell versus (B) fixed cell microscopy images. For the fixed cells, cells were incubated in 4% formaldehyde for 20 min at room temperature. Cell nucleus was stained with Hoechest 33342 (blue). The scale bar is 10 μm.

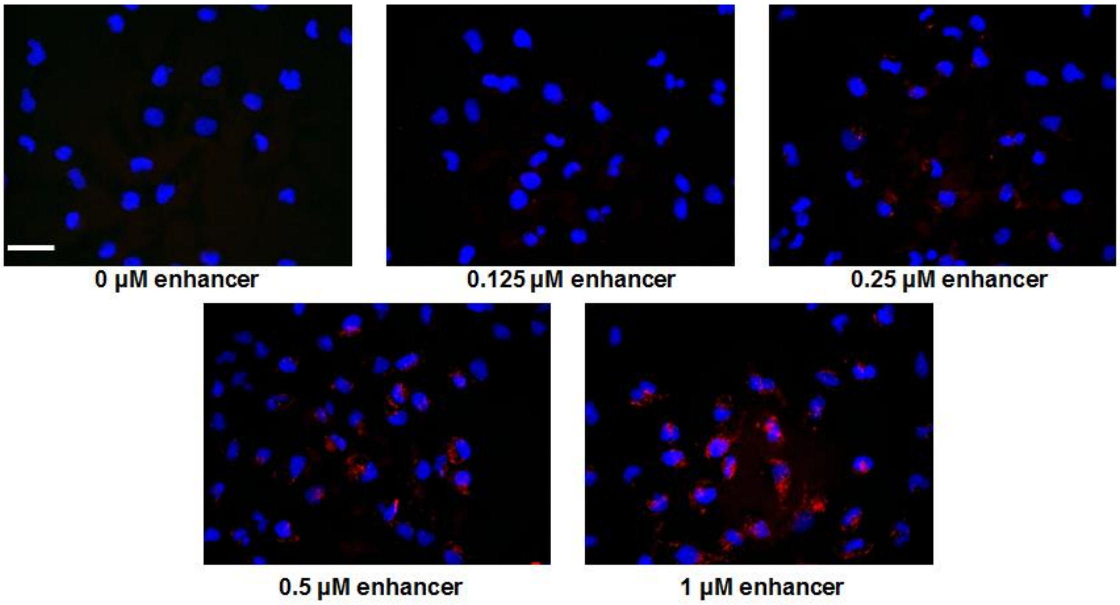

Figure S4. Uptake cooperativity of PNA2 studied by fluoresence microscopy. The Deca-r8-AF-PNA2 (red) was used as the labeled PNA at 0.125 μM in all experiments. The Deca-r8-PNA2 (non-labeled PNA) was added as enhancer at different concentrations. The cells were incubated for 2 h at 37° C after addition of PNAs to the cells. Hoechest 33342 was added for nuclei staining (blue) 10 min before acquiring the images. The scale bar is 30 μm.

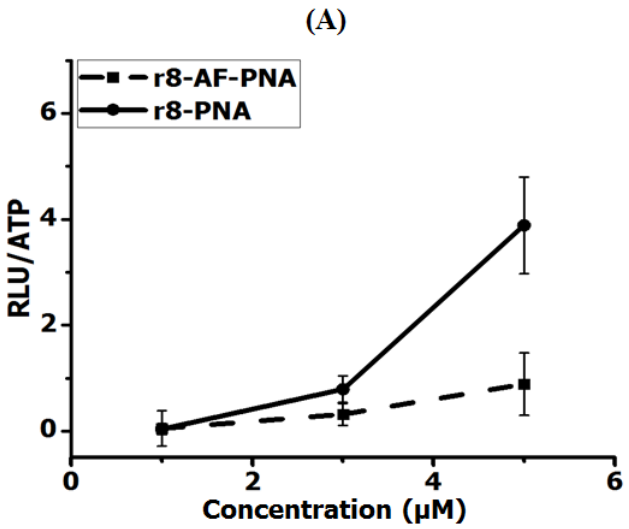

Figure S5. The antisense activity of conjugated PNAs with and without Alexa Fluor in three different concentrations is shown. The transfection time was 20 h and the luciferase activity is normalized by live cells (RLU/ATP).

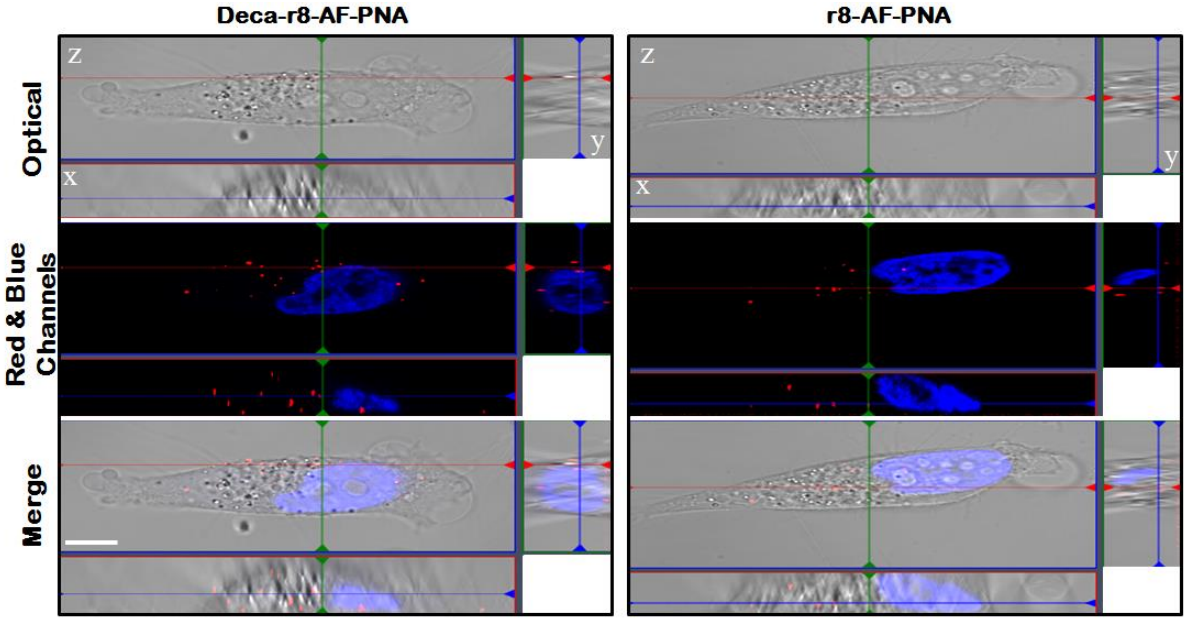

Figure S6. Confocal fluorescence image from cells after treatment with PNA for 2 h. The concentration of Deca-r8-AF-PNA and r8-AF-PNA were 0.125 µM and enhancers (Deca-r8-PNA or r8-PNA) were added at 0.375 µM concentration. Images in three different planes (x, y, and z) are shown to demonstrate that the uptake vesicles are indeed inside the cells. Hoechest 33342 was added to the cells for nuclei staining (blue) 10 min before acquiring the images. The scale bar is 5 µm.

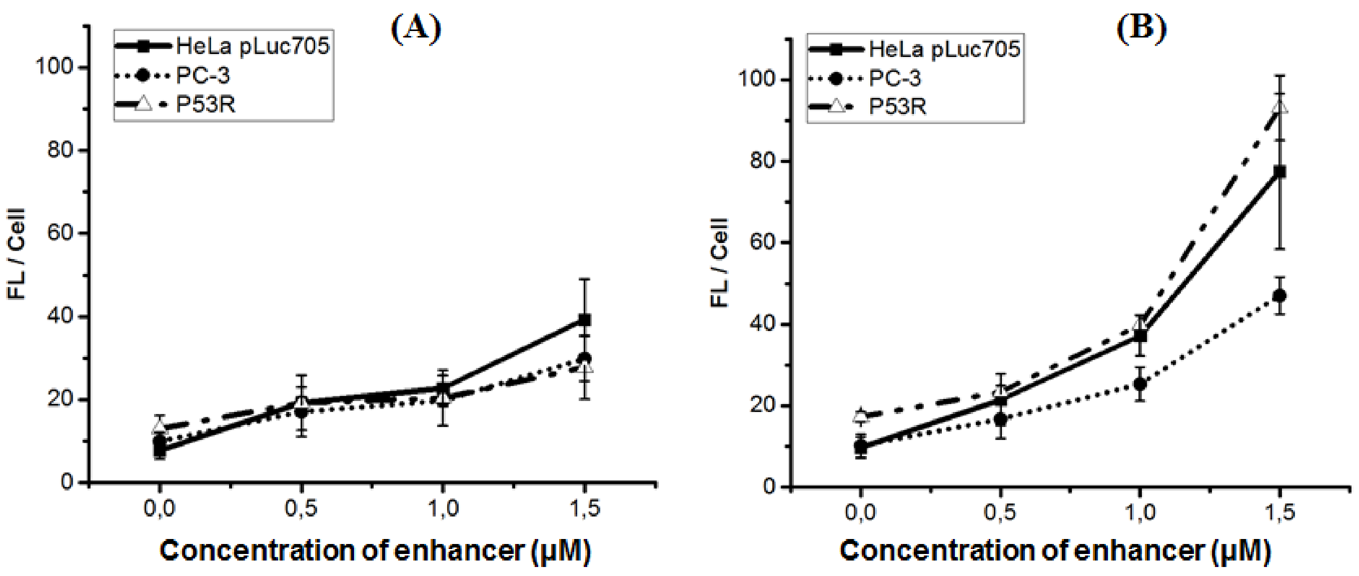

Figure S7. Uptake cooperativity in different cell lines (HeLa pLuc/705, PC-3, and P53R) upon treatment with AF-PNAs for 4 h in the presence of enhancers. The concentration of fluorophore labeled PNA was constant in all experiments and the enhancers were added at different concentrations. The total AF-PNA fluorescence signal measured in each well was normalized to the signal from total DNA of each well (Hoechst 33342). (A) The concentration of r8-AF-PNA was constant at 0.5 μM and the enhancer (r8-PNA) was added at different concentrations. (B) The concentration of Deca-r8-AF-PNA 0.5 μM and the enhancer (Deca-r8-PNA) was added at different concentrations. Each data set represents the mean ± SD of triplicate experiment.

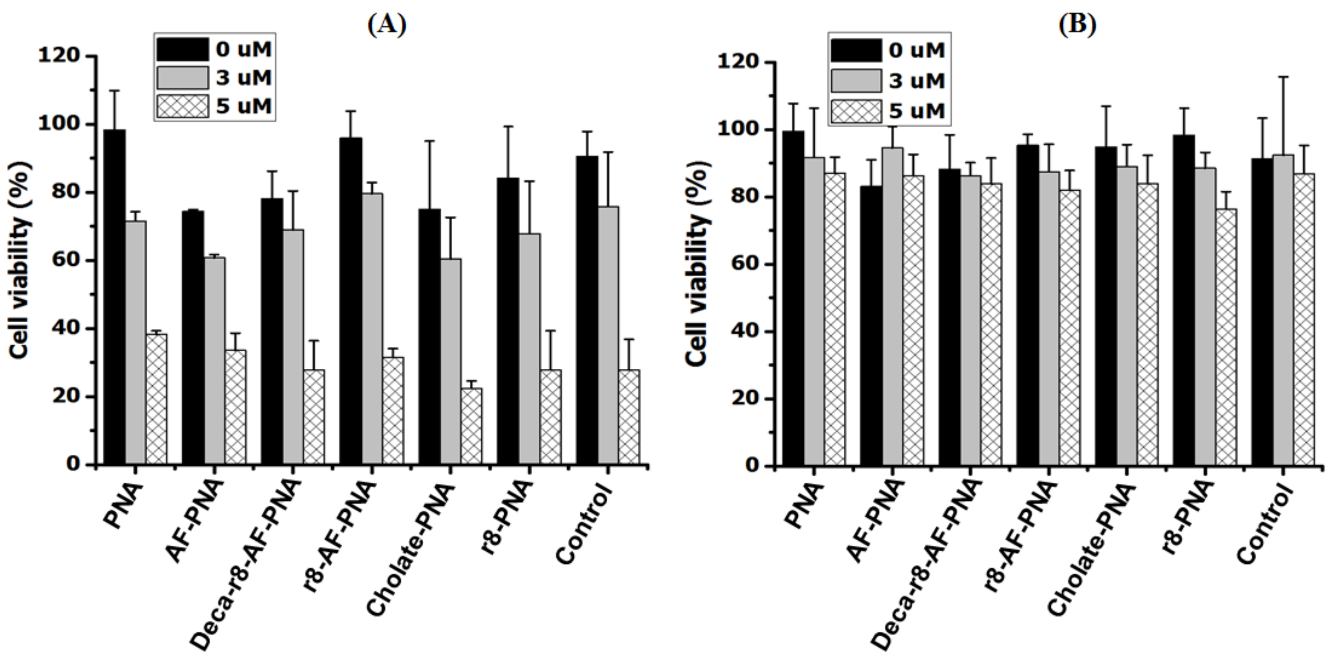

Figure S8. Cytotoxicity of PNAs in the presence of Deca-r8-PNA or Deca-r8 peptide. The different PNAs were used at 1 μM and (A) Deca-r8-PNA_{mm} or (B) Deca-r8 peptide enhancer was added at different concentrations. Cell cultures were incubated for 20 h. Viability was evaluated by measuring ATP level by cell viability assay ATP kit. Each data set represents the mean ± SD of triplicate experiment.

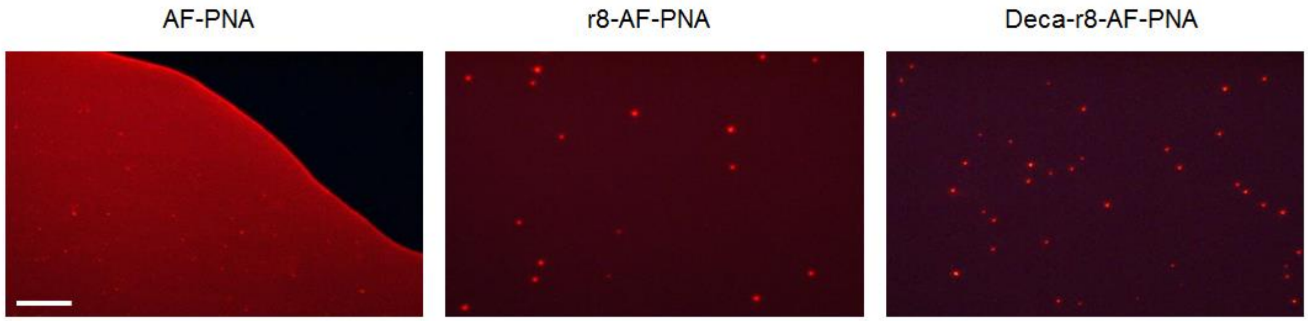

Figure S9. Aggregation of AlexaFluor labeled PNAs in full medium. Different PNAs at 10 μM were prepared in full medium. The fluorescence microscopy images show the formation of particles by r8-AF-PNA and Deca-r8-AF-PNA. The scale bar is 30 μm.

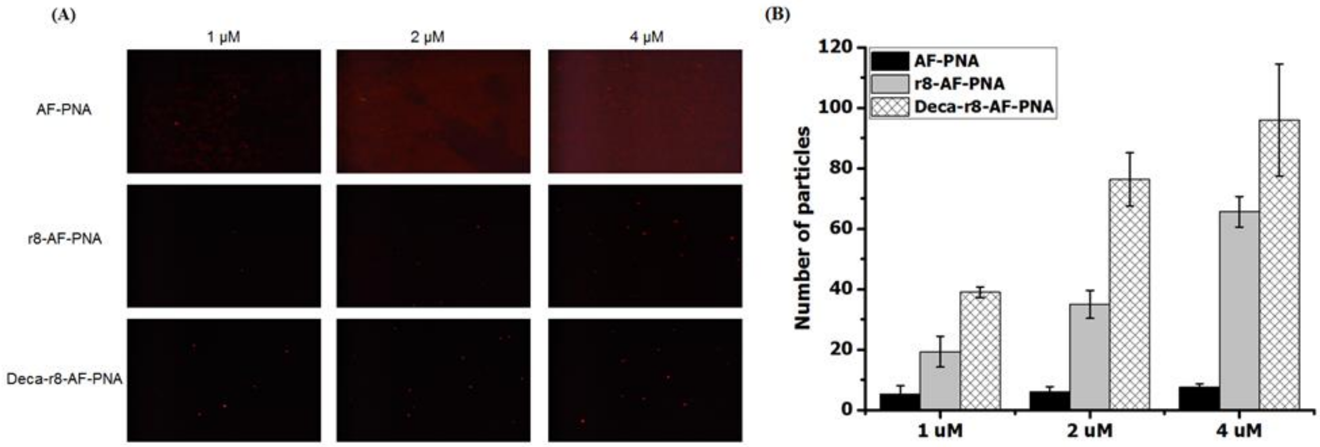

Figure S10. Aggregation of AlexaFluor labeled PNAs in full medium. Different PNAs at three different concentrations were prepared in full medium. The fluorescence microscopy images show the formation of particles by r8-AF-PNA and Deca-r8-AF-PNA (A) and the number of particles is increasing by increasing concentration of PNA (B). The number of particles is calculated by "Fiji Image J" via the function of "analyze particles" with a threshold of 10-100 for all samples. Each data set represents the mean ± SD of triplicate experiment.

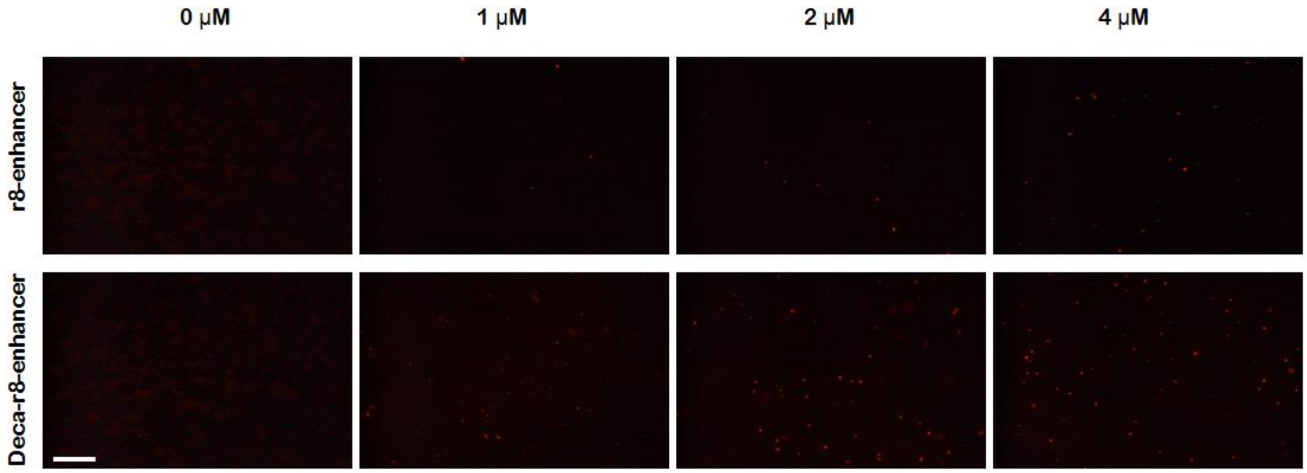

Figure S11. Aggregation of AF-PNAs in full medium in the presence of r8-PNA or Deca-r8-PNA as enhancers. The AF-PNA at the concentration of 1 μM was prepared in full medium in the presence of different concentrations of r8-PNA or Deca-r8-PNA. The fluorescence microscope images clearly show the formation of particles (A) in the presence of r8-PNA and Deca-r8-PNA. The number of particles is increasing by adding more enhancers. The number of particles is calculated by "Fiji Image J" via the function of "analyze particles" with a threshold of 10 -100 for all samples (please refer to Figure 7A). The scale bar is 30 μm.

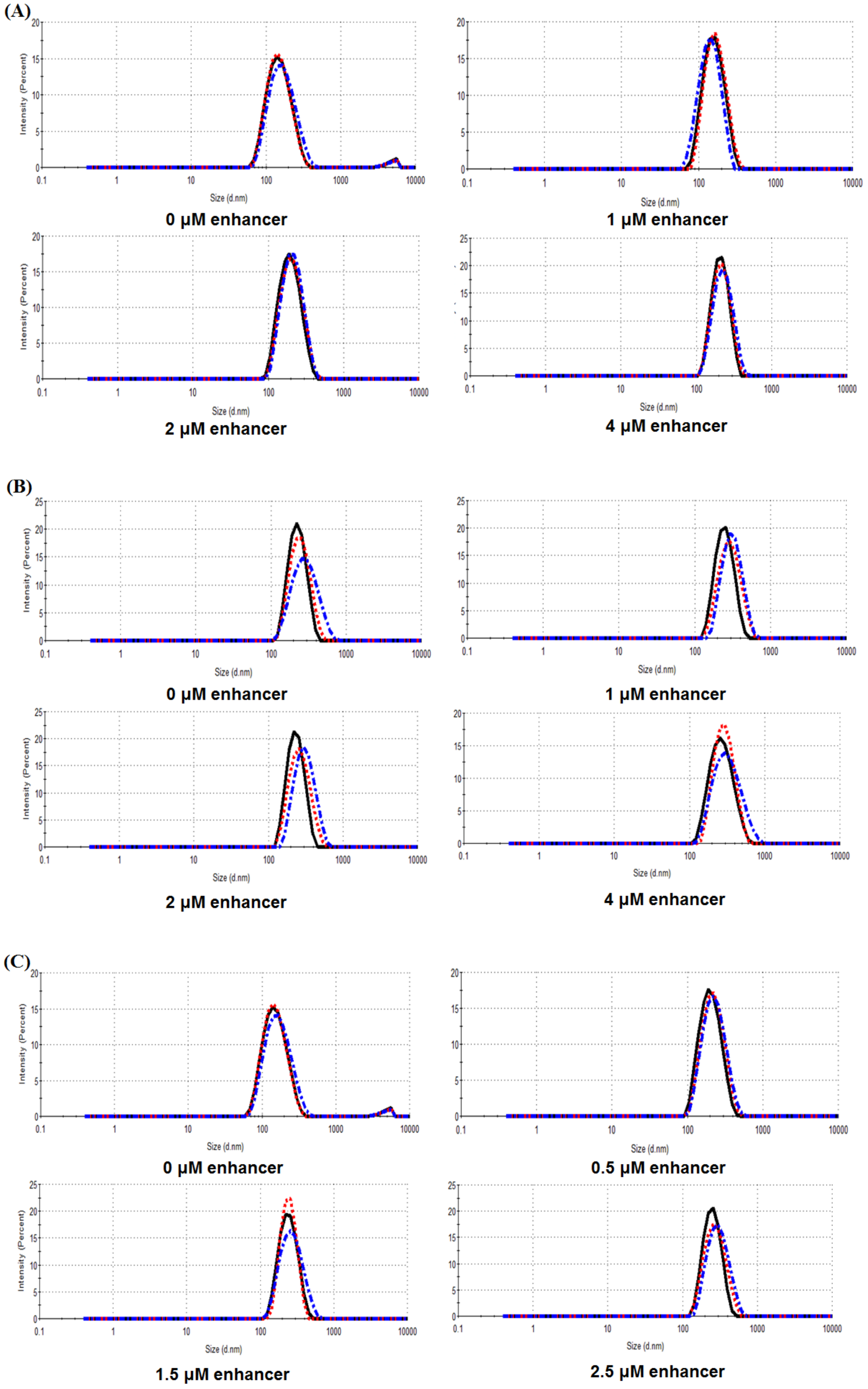

Figure S12. Size characterization of self-assembled nano-aggregations. (A) The r8-PNA at the concentration of 1 μM in the presence of different enhancer (r8-PNA$_{mm}$) in full medium (containing 10% FBS). (B) The AF-PNA at the concentration of 1 μM in the presence of different enhancer (r8-PNA$_{mm}$) in full medium. (C) The r8-PNA at the concentration of 1 μM in the presence of different enhancer (Deca-r8-PNA$_{mm}$) in full medium. Each sample was subjected to three measurements (different colours). The median particle size without enhancer (A) was 150 nm.
